# Subclinical binge eating symptoms in early adolescence and its preceding and concurrent factors: a population-based study

**DOI:** 10.1186/s40337-022-00688-6

**Published:** 2022-11-23

**Authors:** Ivonne P. M. Derks, Holly A. Harris, Soundry Staats, Romy Gaillard, Gwen C. Dieleman, Clare H. Llewellyn, Sonja A. Swanson, Pauline W. Jansen

**Affiliations:** 1 Department of Psychology, Education and Child Studies, Erasmus University Rotterdam, Rotterdam, The Netherlands; 2 Department of Child and Adolescent Psychiatry/Psychology, Erasmus Medical Center, Rotterdam, The Netherlands; 3 Generation R Study Group, Erasmus Medical Center, Rotterdam, The Netherlands; 4 Department of Pediatrics, Erasmus Medical Center, Sophia Children’s Hospital, Rotterdam, The Netherlands; 5 Department of Behavioural Science and Health, University College London, London, UK; 6 Department of Epidemiology, Erasmus Medical Center, Rotterdam, The Netherlands; 7 Department of Epidemiology, University of Pittsburgh, Pittsburgh, PA USA; 8 Erasmus School of Social and Behavioural Sciences, P.O Box 1738, 3000 DR Rotterdam, The Netherlands

**Keywords:** Binge eating, Binge eating disorder, Loss of control, Eating behavior, Prevalence, Adolescence

## Abstract

**Objective:**

Binge eating, loss of control eating and overeating often develop during late childhood or early adolescence. Understanding the presentation of binge eating as early as symptoms manifest and its preceding and concurrent factors is essential to hamper the development of eating disorders. This study examined the prevalence, concurrent and preceding factors (e.g. compensatory behaviors, emotional and behavioral problems) of subclinical binge eating symptoms in early adolescence.

**Methods:**

Data from the population-based Generation R Study were used (n = 3595). At 10 years and 14 years, preceding and concurrent factors including eating behaviors, body dissatisfaction, emotional and behavioral problems and body composition were assessed. At 14 years, 3595 adolescents self-reported on binge eating symptoms in the past 3 months and were categorized into four groups: no symptoms (n = 3143, 87.4%), overeating only (n = 121, 3.4%), loss of control (LOC) eating only (n = 252, 7.0%) or binge eating (i.e. both, n = 79, 2.2%).

**Results:**

In total, 452 (12.6%) young adolescents reported subclinical binge eating symptoms. Those who reported LOC eating and binge eating showed most compensatory behaviors (e.g. hide or throw away food, skipping meals). Concurrent emotional and behavioral problems, body dissatisfaction, more emotional-, restrained- and uncontrolled eating, and a higher BMI were associated with subclinical binge eating symptoms. Preceding self-reported emotional and behavioral problems, body dissatisfaction, more restrained eating and higher BMI (both fat mass and fat-free mass) at 10 years were associated with LOC eating and binge eating, but not with overeating.

**Discussion:**

Among young adolescents, subclinical binge eating symptoms were common. Considering the high prevalence of LOC eating, and the overlapping preceding and concurrent factors of LOC eating and binge eating compared to overeating, LOC eating seems to be a key symptom of binge eating in early adolescence.

**Supplementary Information:**

The online version contains supplementary material available at 10.1186/s40337-022-00688-6.

## Plain English summary

Binge eating (an episode of overeating together with a feeling of loss of control) is a common symptom of most eating disorders and often emerges during late childhood or early adolescence. Examining the presentation of subclinical binge eating symptoms (overeating, loss of control eating and binge eating) during this period and identifying potential risk factors can help to hamper the development of eating disorders. This study in a community sample of young adolescents showed that subclinical binge eating symptoms were common, as these were reported by 12.6% of adolescents, of which loss of control eating only was most common (7%). Unhealthy eating behaviors, poor mental health and higher weight were associated with binge eating symptoms. Prevention strategies may interrupt the development of binge eating by focusing on LOC eating and its risk factors.

**Supplementary Information:**

The online version contains supplementary material available at 10.1186/s40337-022-00688-6.

## Introduction

Binge eating is a shared symptom of many specified eating disorders. Community-based studies of adolescents estimated the prevalence of diagnosed eating disorders to be between 0.3 and 1.7% for Anorexia Nervosa (AN), 0.0–2.6% for Bulimia Nervosa (BN) and 0.0–6.6% for Binge eating Disorder (BED) [1–5]. Subthreshold or subclinical eating disorders, in which the frequency of binge eating episodes or other symptoms is lower (for instance, once per month), are also common in adolescence, estimated at 0.8% for AN, 4.4% for BN and 0.2–18.5% for BED [1, 2, 5]. Adolescents who experience binge eating episodes are at risk for adverse physical and psychiatric outcomes later in life, including obesity, anxiety, depression, substance use, self-harm and suicidality [1, 6–8].The two components of binge eating (i.e. overeating and loss of control (LOC) eating) have also been studied separately and showed that LOC eating may be a more debilitating component of binge eating compared to overeating [9]. In (pre-)adolescence, LOC eating is shown to be a common, moderately stable disordered eating behavior linked to eating disorder psychopathology [10–12], overweight and obesity [13, 14], poorer mental health and emotion regulation [12, 15].

Binge eating, LOC eating and overeating may already emerge in late childhood or early adolescence with puberty reflecting a critical period [1, 16, 17]. Understanding the presentation of binge eating as early as symptoms manifest, as well as knowledge about its predictors is essential to inform screening, prevention and intervention strategies that aim to avert the adverse consequences associated with binge eating. Previously identified predictors of binge eating episodes may include overweight/obesity, emotional and behavioral problems/disorders, negative affect and body dissatisfaction [13, 18–21]. Other studies showed that childhood bullying and Attention Deficit Hyperactivity Disorder are associated with binge eating [22, 23]. Eating behaviors during childhood may also be predictive of binge eating in adolescence, including eating in the absence of hunger, overeating and a strong desire for food [4, 23, 24]. Likewise, a number of potentially modifiable risk factors for LOC eating have been proposed, including weight-based teasing, depressive and anxiety symptoms, emotion dysregulation, impulsivity, negative affect and low emotional awareness [15, 25–27]. Studies also compared potential risk factors of overeating compared to binge eating and showed that engagement in weight control behaviors, decreased self-esteem, body dissatisfaction and more depressive symptoms were more associated with binge eating relative to overeating [28, 29]. Inconsistent findings have been reported regarding the role of socioeconomic status and ethnic background in binge eating [1, 21, 30, 31]. Furthermore, a study showed that parents with BED reported more binge eating in their children [32], although other research did not observe such an association for a wider range of parental psychopathology [21].

The previous studies on potential risk factors for overeating, LOC eating and binge eating were often cross-sectional [1, 18, 20, 21, 25, 26, 28, 32], conducted in samples of adolescents with overweight/obesity, high-risk groups or girls alone [13, 15, 19, 24, 27] or examined relationships in late adolescence [15]. This highlights the need for prospective population-based studies on binge eating in early adolescence. Therefore, this study aimed to estimate the prevalence of subclinical binge eating symptoms (overeating only, LOC eating only, or binge eating) in a large community-based sample of adolescents with a mean age of 14 years. Furthermore, we described the association of a range of concurrent and preceding factors, including compensatory behaviors, demographic and parental characteristics, other disordered eating behaviors, mental health problems and weight status, with subclinical binge eating symptoms.

## Methods

### Study population

This study was embedded in the Generation R Study, a population-based prospective cohort. The design has been described previously [33]. In brief, all pregnant women living in Rotterdam, the Netherlands, with an expected delivery date between April 2002 and January 2006 were invited to participate (participation rate = 61%, total n live births = 9747). The Medical Ethical Committee of the Erasmus Medical Center approved the study. In the 14-year wave, 6842 adolescents consented to participate. Adolescents were included in the current study if they reported on binge eating symptoms (n = 3595), and were categorized into four groups: adolescents who experienced no binge eating symptoms (n = 3143), overeating only (n = 121), LOC eating only (n = 252), and binge eating (n = 79) in the past 3 months.

Compared to those without information on subclinical binge eating symptoms (n = 3247), adolescents included in the study sample were more often girls, more often of Dutch ethnicity, had a higher household income and lower BMI. Moreover, mothers reported lower behavioral problems, but not emotional problems, for adolescents included in the study sample (see Additional file 1: Table S1 for comparative data).

### Measurements

#### Subclinical binge eating symptoms

Binge eating symptoms were assessed by a self-report questionnaire at age 14 years. Items were based on the Developmental and Well-Being Assessment (DAWBA) [34, 35], an instrument for DSM-5 diagnoses with good psychometric properties for eating disorders [36]. First, a description of an overeating episode was provided: “Sometimes people eat a very large amount of food in a very short time. For example, they may open the fridge and eat as much as they can find, eating and eating until they feel physically ill. This usually happens when people are by themselves. Does this happen to you? (yes/no)”. If “yes”, they were asked “How often did this happen on average in the past 3 months?” with four responses ranging from “Did not happen” to “Once or more per week”. Second, LOC eating was measured by asking: “In the past 3 months, has there been a time when you were eating and you felt like you couldn’t stop? During which you just kept eating and eating and couldn’t stop even if you wanted to? (yes/no)” If yes, they were asked “How often did this happen on average in the past 3 months?” Again, responses ranged from “Did not happen” to “Once or more per week”. Item frequencies for the total sample and stratified by adolescent’s sex are reported in Additional file 1: Table S2.

Adolescents were categorized into groups of subclinical binge eating symptoms according to the occurrence and frequency of overeating and LOC eating symptoms. Adolescents who reported no overeating and LOC eating in the past 3 months were categorized in the ‘no symptoms’ group. Adolescents who reported to have experienced overeating in the past 3 months (less than once per month; one or more times per month; one or more times per week), but not LOC eating, were categorized in the ‘overeating’ group. Likewise, the ‘LOC eating’ group consisted of adolescents who experienced LOC eating but not overeating in the past 3 months. Adolescents were categorized into the ‘binge eating’ group when they reported overeating and LOC eating in the past 3 months.

A similar categorization of groups was made which presents more frequent occurrence of symptoms; here, the categorization was based on whether adolescents reported weekly binge eating symptoms, which aligns with the clinical DSM-5 criteria to diagnose BED or BN (for a diagnosis, symptoms have to be present weekly for at least 3 months). The weekly categorization was not used in further analyses.

#### Compensatory behaviors

Compensatory behaviors were also assessed by self-reported questionnaire at 14 years with the DAWBA [34, 35]. The frequency of eight compensatory behaviors was assessed by asking “In the past 3 months, did you do any of the following behaviors in order to lose weight or to prevent weight gain?” The compensatory behaviors assessed were: eat less during meals, meal skipping, not eating for a whole day or a large part of the day, hide or throw away food that others gave you, avoidance of high-caloric food, increased exercise, purging, and the use of medications that contribute to weight loss. Four response options were provided, ranging from “No” to “Once per week”. From this, a binary variable was created for each compensatory behavior separately: the compensatory behavior occurred (based on answering options “once per week” and “once per month”) or not (“seldom” and “no”).

#### Demographic and parental characteristics

Adolescent’s *sex* and date of birth, from which *age at outcome assessment* was calculated, were derived from midwife- or hospital registries. Adolescent’s *ethnicity* was derived from the country of birth of both parents (Dutch; Other Western; Non-Western), that was assessed by prenatal questionnaire. Mothers reported on their net monthly *household income* when children were 6 years old (less than 2000 euro; between 2000 and 4000 euro; more than 4000 euro per month). In the same questionnaire, mothers reported on their highest obtained *education level* (no education to secondary education; lower vocational education/higher vocational education; university). Maternal height and weight were measured in the first trimester of pregnancy, from which *maternal BMI* was calculated. Paternal height and weight were assessed by self-report in the prenatal questionnaire, from which *paternal BMI* was calculated. Mothers reported on *lifetime history of eating disorders* (AN and BN, according to the current DSM IV at that time) during pregnancy and when their child was 10 years old (yes; no). When mothers reported “yes” on either AN or BN at one or both time points, they were classified as having a history of eating disorders. Finally, *maternal and paternal depressive symptoms* were assessed when the adolescent was 10 years old with a short version of the Brief Symptom Inventory (BSI) [37].The depression subscale consists of 6 items with four answering options from “not at all” to “extremely”, from which a weighted score was calculated.

#### Concurrent factors

 At the age of 14 years, adolescents self-reported on a range of eating behaviors. *Emotional* (3 items) and *uncontrolled eating* (4 items) were measured with the Three Factor Eating Questionnaire (TFEQ-R18) [38, 39]. Item responses were on a 4-point Likert scale (“definitely false” to “definitely true”). *Restrained eating* (10 items) was measured with the Dutch Eating Behavior Questionnaire (DEBQ) [40]. Responses were on a 4-point Likert scale (“never” to “once or more times per week”). For all eating behaviors, items were summed and weighted, allowing for 25% missing items. *Body dissatisfaction* was assessed using body figure perceptions in line with the Children’s Body Image Scale [41, 42], which consisted of seven gender-specific adolescent figures with increasing adiposity levels. Body dissatisfaction was calculated from two questions following the figures: (1) “Which body is most like you?” and (2) “Which body would you like to have?”. Body size satisfaction was calculated as the difference between the two questions, with scores ranging from − 6 to 6, that were collapsed into two categories: the desire to be thinner, versus the rest (adolescents who were satisfied and adolescents who desired to be heavier). Emotional and behavioral problems were assessed using the Youth Self Report (YSR), a 112-item questionnaire anchored on a 3-point Likert scale (not true; sometimes true; often true) [43–45]. Sum scores of the two broadband scales ‘internalizing’ and ‘externalizing’ that reflect *emotional* and *behavioral problems*, respectively, were used in this study. Mothers also reported on their *adolescent’s emotional* and *behavioral problems*, by reporting on the Child Behavior Checklist (CBCL), 6–18 year version [46]. Adolescent’s BMI was calculated from height and weight measured during the 14 years visit and transformed into sex- and age adjusted standardized *BMI scores* using Dutch reference growth curves [47].

#### Preceding factors

All preceding factors were assessed when participants were 10 years old. Children self-reported on their problem behavior, using the Brief Problem Monitor, Youth version [48], comprising of 19 items scored on a 3-point Likert scale (not true; sometimes true; often true). Again, sum scores of the two scales internalizing (6 items) and externalizing (7 items), reflecting *emotional problems* and *behavioral problems* were used. Next to that, children reported on their body satisfaction, again using the validated Child Body Image Scale and using the same approach as age 14 years, only now with the child-version of the gender-specific figures (41). Mothers reported on their child’s eating behaviors with the Child Eating Behavior Questionnaire (35-items) [49–52]. Higher scores on *emotional overeating* (4 items), *enjoyment of food* (4 items) and *food responsiveness* (4 items) are considered to reflect food approach behaviors, while higher scores on *satiety responsiveness* (5 items) reflects food avoidant eating behavior. Item responses were on a 5-point Likert scale from “never” to “always” and weighted sum scores were calculated, allowing for 25% missing per subscale. Mothers reported on children’s *restrained eating* behavior using the parent-reported version of the DEBQ [53], consisting of 9 items with responses on a 5-point Likert scale, ranging from “never” to “very often”. *Mother-reported emotional and behavioral problems* were assessed with the CBCL [46]. Sex- and age- adjusted standardized *BMI scores* were calculated after assessment of height and weight at the 10 year visit, again using the Dutch reference growth curves [47]. Body composition was measured using a Dual-Energy-X-ray-Absorptiometry (DXA) scanner. Based on all participating children with body composition measures available, sex- and age adjusted *Fat Mass Index (FMI)* and *Fat Free Mass Index (FFMI)* were calculated as fat (free) mass (kg)/height (m)^2^.

### Statistical analyses

First, the prevalence of compensatory behaviors by subgroups of binge eating symptoms was studied. Descriptive demographic and parental characteristics were reported for the total study sample and by binge eating subgroup. 95% confidence intervals were obtained with bootstrapping of the point estimates, for which results of 1000 samples are presented.

Second, for each concurrent or preceding factor, sum scores were standardized for comparison purposes. Cross-sectional associations between concurrent factors at 14 years and subclinical binge eating symptom subgroups were examined using multinomial logistic regression analyses. Likewise, associations between preceding factors at 10 years and subclinical binge eating at 14 years were studied using multinomial logistic regression analyses. All analyses were adjusted for adolescent’s biological sex and exact age at reported binge eating symptoms. We further explored how these associations varied by the adolescent’s sex, by adding product terms to the above-described models. If the association was moderated by adolescent’s sex, differences were examined by stratified analyses.

Because of the explorative nature of the study, each analysis was based on available data. Therefore, cases were excluded if there was missing information on each of the variables for every analysis separately.

## Results

### Prevalence of subclinical and clinical binge eating symptoms

In total, 12.6% of the adolescents reported subclinical binge eating symptoms in the past 3 months (Table 1). Of those, 3.4% reported overeating only, 7.0% reported LOC eating only and 2.2% reported binge eating. Weekly binge eating symptoms were also reported by adolescents but were comparatively rare (weekly overeating and LOC eating: 0.6%, weekly binge eating: 0.1%).

**Table 1:** Frequency of subclinical and clinical binge eating symptoms in the past 3 months in 14 year old adolescents (n = 3595)

	Binge eating symptoms			
	No symptoms	Overeating only	LOC eating only	Binge eating (overeating and LOC eating)
	n (%)	n (%)	n (%)	n (%)
Total				
≥ 1 in the past 3 months	3143 (87.4)	121 (3.4)	252 (7.0)	79 (2.2)
≥ 1 times per week in past 3 months	3546 (98.6)	22 (0.6)	22 (0.6)	5 (0.1)
Males				
≥ 1 in the past 3 months	1525 (89.8)	47 (2.8)	104 (6.1)	23 (1.4)
≥ 1 times per week in past 3 months	1680 (98.9)	6 (0.4)	10 (0.6)	3 (0.2)
Females				
≥ 1 in the past 3 months	1618 (85.3)	74 (3.9)	148 (7.8)	56 (3.0)
≥ 1 times per week in past 3 months	1866 (98.4)	16 (0.8)	12 (0.6)	2 (0.1)

*≥1 in times per week in past 3 months can be considered as clinical, ≥ 1 times in past 3 months can be considered as subclinical. *LOC* loss of control

#### Compensatory behaviors

Compared to no symptoms, adolescents with overeating reported that they exercise more frequently (Table 2). Adolescents with LOC eating and binge eating, compared to no symptoms, more often reported to avoid eating all or most of the day, to hide or throw away food, to exercise more, to eat less during meals, to skip meals and to avoid fattening foods. The highest frequencies of compensatory behaviors were observed in the binge eating group, followed by LOC eating, although confidence intervals overlapped.

**Table 2:** Prevalence of adolescents (14 years) engaging in compensatory behavior by binge eating symptoms in the past 3 months (n = 3595)

Compensatory behaviors, at least one time per month	Binge eating symptoms			
	No symptoms	Overeating only	LOC eating only	Binge eating (overeating and LOC eating)
	n = 3143 % (95% CI)	n = 121 % (95% CI)	n = 252 % (95% CI)	n = 79 % (95% CI)
Purging	0.2 (0.0, 0.3)	0.8 (0.0, 2.6)	0.4 (0.0, 1.2)	3.8 (0.0, 8.6)
Use of weight loss medications	0.0 (0.0, 0.1)	0.0 (0.0, 0.0)	0.0 (0.0, 0.0)	0 (0.0, 0.0)
Don’t eat all day or most of the day	3.7 (3.0, 4.3)	5.9 (1.7, 10.3)	14.0 (9.6, 18.0)	15.2 (7.5, 22.8)
Hide or throw away food	1.6 (1.2, 2.0)	4.2 (0.8, 8.3)	7.2 (4.0, 10.4)	12.7 (6.3, 20.5)
Exercise more	26.8 (25.3, 28.3)	43.3 (34.7, 52.5)	42.5 (35.8, 48.8)	55.1 (44.0, 66.7)
Eat less during meals	7.8 (6.9, 8.7)	15.0 (8.5, 21.3)	18.7 (14.3, 23.8)	39.2 (29.1, 50.0)
Skip meals	3.9 (3.3, 4.7)	7.5 (3.3, 12.7)	12.3 (8.4, 16.5)	26.6 (16.5, 36.3)
Avoid food that makes you fat	15.5 (14.3, 16.8)	16.7 (10.0, 23.1)	30.2 (24.7, 35.7)	41.8 (31.6, 53.1)

*Missings for compensatory behaviors ranged from 16 (purging) to 52 (exercise more). 95% confidence intervals were obtained by using bootstrapping (results of 1000 samples). *LOC* loss of control, *CI* confidence Interval

#### Demographic and parental characteristics

Adolescents with binge eating symptoms were older than those with no symptoms (binge eating: 14.08 years, 95% CI 13.91, 14.23 vs. no symptoms: 13.87 years, 95% CI 13.85, 13.89), and more often girls (binge eating: 70.9%, 95% CI 60.3, 80.8, vs. no symptoms: 51.5%, 95% CI 49.7, 53.2) (Table 3). Compared to mothers of adolescents without symptoms, mothers of adolescents with LOC eating more often had a history of eating disorders (LOC eating: 14.8%, 95% CI 10.4, 19.2 versus no symptoms: 8.8%, 95% CI 7.7, 9.8) and had a higher pre-pregnancy BMI. No other differences were observed.

**Table 3:** Demographic and parental characteristics of young adolescents by binge eating symptoms in the past 3 months (n = 3595)

Demographic and parental characteristics	Total n = 3595	Binge eating symptoms			
		No symptoms	Overeating only	LOC eating only	Binge eating (overeating and LOC eating)
		n = 3143	n = 121	n = 252	n = 79
Age at outcome assessment (years), mean (95% CI)	3595	13.87 (13.85, 13.89)	13.97 (13.86, 14.09)	13.94 (13.86, 14.02)	14.08 (13.91, 14.23)
Sex, % (95% CI)					
Girls	1896	51.5 (49.7, 53.2)	61.2 (52.9, 69.7)	58.7 (52.6, 64.9)	70.9 (60.3, 80.8)
Boys	1699	48.5 (46.8, 50.3)	38.8 (30.3, 47.5)	41.3 (35.3, 47.4)	29.1 (19.2, 39.2)
Ethnicity, % (95% CI)					
Dutch	2444	68.5 (66.9, 70.2)	73.6 (65.6, 81.1)	67.1 (60.9, 72.5)	65.8 (55.0, 76.5)
Other western	313	8.9 (7.9, 9.9)	6.6 (3.2, 11.5)	8.4 (5.2, 12.0)	8.9 (2.5, 15.6)
Non-western	809	22.6 (21.1, 24.0)	19.8 (12.6, 27.0)	24.5 (19.4, 30.1)	25.3 (15.4, 35.4)
Household income, % (95% CI)					
High (> 4000 euros per month)	1292	40.5 (38.5, 42.4)	35.8 (26.9, 45.3)	41.5 (35.0, 48.6)	44.4 (31.7, 57.4)
Medium (1600–4000 euros per month)	1587	50.0 (48.0, 52.0)	55.7 (46.7, 65.4)	46.7 (39.6, 53.3)	39.7 (28.1, 52.4)
Low (< 1600 euros per month)	312	9.5 (98.4, 10.4)	8.5 (3.7, 13.8)	11.8 (7.4, 16.1)	15.9 (7.8, 25.0)
*Parental characteristics*					
Maternal educational level, % (95% CI)					
High (higher vocational education to university)	2195	66.3 (64.6, 68.0)	63.7 (54.9, 72.8)	62.8 (56.4, 69.2)	63.2 (51.4, 74.6)
Medium (lower vocational education)	880	26.4 (24.9, 27.9)	30.1 (21.3, 38.7)	26.0 (20.3, 31.8)	25.0 (15.7, 36.2)
Low (no education to high school)	254	7.3 (6.3, 8.3)	6.2 (2.6, 10.6)	11.2 (7.6, 15.6)	11.8 (4.5, 19.1)
Maternal history of an eating disorder, % (95% CI)					
Yes	319	8.8 (7.7, 9.8)	13.4 (7.6, 19.8)	14.8 (10.4, 19.2)	7.8 (2.6, 13.9)
No	3098	91.2 (90.2, 92.3)	86.6 (80.2, 92.4)	85.2 (80.8, 89.6)	92.2 (68.1, 97.4)
Maternal pre-pregnancy BMI, mean (95% CI)	3192	24.21 (24.05, 24.36)	24.93 (24.20, 25.68)	25.01 (24.46, 25.63)	25.00 (24.10, 25.96)
Paternal BMI during pregnancy, mean (95% CI)	2732	25.15 (25.01, 25.29)	25.41 (24.83, 25.99)	25.53 (25.03, 26.09)	25.93 (25.07, 26.77)
Maternal depressive symptoms, median (95% CI)	3138	0.00 (0.00, 0.00)	0.00 (0.00, 0.17)	0.00 (0.00, 0.17)	0.00 (0.00, 0.17)
Paternal depressive symptoms, median (95% CI)	2600	0.00 (0.00, 0.00)	0.00 (0.00, 0.00)	0.00 (0.00, 0.00)	0.00 (0.00, 0.08)

*Missings for demographic characteristics ranged from 0 to 873 (paternal BMI). 95% confidence intervals were obtained by using bootstrapping (results of 1000 samples). *LOC* loss of control, *CI* confidence Interval

#### Concurrent factors

Adolescents with more emotional overeating, uncontrolled eating and restrained eating had higher odds of being in the overeating, LOC eating or binge eating group as compared to adolescents without symptoms (for instance, adolescents with an 1 SD increase in emotional overeating score had a 2.25 odds of being in the binge eating subgroup, 95% CI 1.97, 2.57) (Table 4). Given the overlap in content of measures, these associations can be interpreted as a validation of the binge eating subgroups. Adolescents who desired to be thinner had an increased odds of overeating, LOC eating and binge eating (e.g. binge eating: OR = 4.75, 95% CI 2.93, 7.69). More self-reported emotional and behavioral problems were associated with all subclinical binge eating symptoms. Similar but smaller associations were observed with mothers-reported emotional and behavioral problems. Finally, a higher BMI was associated with subclinical binge eating symptoms. For example, adolescents with a 1 SD higher BMI had a 1.39 odds of being in the LOC eating subgroup (95% CI 1.22, 1.58).

**Table 4:** Univariate associations between concurrent factors and binge eating symptoms in the past 3 months in early adolescence (n = 3595)

Concurrent factors at 14 years	Total n	Binge eating symptoms at 14 years			
		No symptoms	Overeating only	LOC eating only	Binge eating (overeating and LOC eating)
			OR (95% CI)	OR (95% CI)	OR (95% CI)
Self-reported					
Emotional overeating	3570	Ref.	1.66 (1.45, 1.90)	1.69 (1.53, 1.87)	2.25 (1.97, 2.57)
Uncontrolled eating	3580	Ref.	2.24 (1.89, 1.66)	2.97 (2.61, 3.37)	5.49 (4.44, 6.80)
Restrained eating	3574	Ref.	1.28 (1.09, 1.51)	1.49 (1.34, 1.66)	1.87 (1.57, 2.22)
Body dissatisfaction	3439	Ref.	1.53 (1.03 2.26)	2.17 (1.66, 2.85)	4.75 (2.93, 7.69)
Emotional problems	3476	Ref.	1.34 (1.13, 1.59)	1.59 (1.42, 1.79)	2.24 (1.89, 2.64)
Behavioral problems	3467	Ref.	1.72 (1.47, 2.01)	1.54 (1.37, 1.75)	2.10 (1.77, 2.50)
Mother-reported					
Emotional problems	3458	Ref.	1.04 (0.86, 1.26)	1.20 (1.06, 1.35)	1.37 (1.14, 1.65)
Behavioral problems	3452	Ref.	1.47 (1.26, 1.72)	1.21 (1.06, 1.38)	1.69 (1.43, 1.99)
Body composition					
BMI SD score	3286	Ref.	1.34 (1.13, 1.59)	1.39 (1.22, 1.58)	1.59 (1.28, 1.98)

*All concurrent factors were standardized. Results were obtained with multinomial logistic regression analysis and all associations were adjusted for adolescent sex and exact age at outcome assessment. *LOC* loss of control, *OR* odds ratio, *CI* confidence Interval, *SD* standard deviation

#### Preceding factors

More self-reported emotional and behavioral problems at 10 years were associated with a higher odds of LOC eating and binge eating, but not overeating (Table 5). For instance, 1 SD increase in emotional problems increased the odds of subclinical binge eating by 67% (OR = 1.67, 95% CI 1.35, 2.06). Body dissatisfaction was associated with an increased odds of LOC eating and binge eating, but not overeating. Mother-reported emotional overeating and food responsiveness were positively associated with all subclinical binge eating symptoms at age 14, whereas mother-reported enjoyment of food was positively associated with LOC eating, but not overeating and binge eating. High satiety responsiveness was associated with a lower odds of LOC eating and binge eating (for example, per 1 SD increase in satiety responsiveness score: OR _binge eating_= 0.73, 95% CI = 0.56, 0.95). Mother-reported emotional problems increased the odds of LOC eating, while behavioral problems increased the odds of overeating (OR = 1.25, 95% CI = 1.07, 1.46). Finally, restrained eating, higher BMI, FMI and FFMI were all associated with an increased odds of LOC eating and binge eating, but not overeating.

**Table 5:** Univariate associations between preceding factors at 10 years and binge eating symptoms in the past 3 months at 14 years (n = 3595)

Preceding factors at 10 years	Total n	Binge eating symptoms at 14 years			
		No symptoms	Overeating only	LOC eating only	Binge eating (overeating and LOC eating)
			OR (95% CI)	OR (95% CI)	OR (95% CI)
Self-reported					
Emotional problems	2995	Ref.	1.13 (0.93, 1.37)	1.33 (1.17, 1.51)	1.67 (1.35, 2.06)
Behavioral problems	2986	Ref.	1.19 (0.99, 1.43)	1.18 (1.03, 1.35)	1.36 (1.09, 1.70)
Body dissatisfaction	2975	Ref.	1.13 (0.74, 1.73)	1.91 (1.43, 2.56)	2.46 (1.49, 4.07)
Mother-reported					
Emotional overeating	3124	Ref.	1.23 (1.04, 1.46)	1.39 (1.24, 1.57)	1.30 (1.05, 1.61)
Food responsiveness	3189	Ref.	1.31 (1.10, 1.55)	1.53 (1.36, 1.72)	1.70 (1.40, 2.06)
Enjoyment of food	3193	Ref.	1.06 (0.88, 1.29)	1.28 (1.11, 1.48)	1.22 (0.95, 1.57)
Satiety responsiveness	3196	Ref.	0.99 (0.82, 1.20)	0.87 (0.76, 1.00)	0.73 (0.56, 0.95)
Restrained eating	3182	Ref.	1.01 (0.83, 1.22)	1.27 (1.13, 1.43)	1.27 (1.03, 1.56)
Emotional problems	3157	Ref.	1.17 (0.99, 1.39)	1.15 (1.01, 1.30)	1.07 (0.84, 1.37)
Behavioral problems	3159	Ref.	1.25 (1.07, 1.46)	1.13 (0.99, 1.28)	1.20 (0.96, 1.49)
Body composition					
BMI SD score	3329	Ref.	1.19 (0.99, 1.43)	1.34 (1.17, 1.54)	1.72 (1.36, 2.19)
Fat Mass Index SD score	3294	Ref.	1.14 (0.93, 1.40)	1.35 (1.18, 1.54)	1.60 (1.29, 1.98)
Fat Free Mass Index SD score	3294	Ref.	1.17 (0.96, 1.42)	1.24 (1.08, 1.43)	1.53 (1.22, 1.92)

*All preceding factors were standardized. Results were obtained with multinomial logistic regression analysis and all associations were adjusted for adolescent sex and exact age at outcome assessment. *LOC* loss of control, *OR* odds ratio, *CI* confidence interval, *SD* standard deviation

#### Differences by sex

Stratified results by sex are presented in Additional file 1: Table S3, but only when an interaction effect by sex was observed in the association. For concurrent factors, girls with more restrained eating and self-reported behavioral problems had a higher odds of binge eating at 14 years, compared to boys (e.g. Girls: OR_restrained eating−binge eating_= 2.23 95% CI 1.81, 2.74, Boys: OR_restrained eating−binge eating_= 1.12 95% CI 0.73, 1.71). For preceding factors, girls with a higher BMI, FMI or FFMI at 10 years had a higher odds of binge eating as compared to boys.

## Discussion

This study examined the prevalence, concurrent and preceding factors of subclinical binge eating symptoms in a population-based sample of 14-year old adolescents. Our findings show that, overall, subclinical binge eating symptoms were common in young adolescents, as 12.6% of the sample experienced either overeating, LOC eating or binge eating at least once in the past 3 months. However, the prevalence of weekly (clinical) and three-monthly (subclinical) binge eating (i.e. a combination of overeating and LOC eating), 0.1% and 2.2% respectively, was lower compared to most other studies reporting on (sub)clinical binge eating symptoms in adolescence. A recent meta-analysis in community and clinical samples of adolescents (aged 12–20 years) reported a prevalence of 1.39% for BED and 3.00% for subclinical BED [5]. The studies included in this meta-analysis used more stringent definitions for subclinical binge eating, by excluding binge eating symptoms in the context of other eating disorders (for instance, combined with compensatory behaviors). Therefore, the prevalence of binge eating symptoms may even be underestimated in these studies. However, in a more comparable British community sample the prevalence of weekly binge eating – regardless of compensatory behaviors – was also higher: 3.9% of boys and 15.8% of girls experienced binge eating at the age of 16 years [4]. This difference in prevalence might be explained by the younger age of our study sample, as binge eating may occur more frequently with age. This was also demonstrated in the current study, where adolescents with binge eating were slightly older compared to adolescents with no symptoms.

We showed that LOC eating was more prevalent than overeating and binge eating, with a prevalence of 7.0% in the past 3 months. Other studies did not directly compare the prevalence of LOC eating with overeating and binge eating. One study in adolescents aged 12–20 years (mean age = 15 years) reported that 23.3% experienced any LOC eating in the past 28 days. However, of those, many met the criteria for (subthreshold) BED and thus experienced overeating as well [10]. Furthermore, in children and adolescents with overweight/obesity, LOC eating occurred more often than binge eating in the past 3 months, namely 31.2% compared to 22.2% [13]. But again, overeating was not examined. So far, this suggests that LOC eating only is frequently present among youth, and may be more common than overeating or binge eating, perhaps because LOC eating is a more cognitive and subjective feeling of overeating that can occur without actually eating an objectively large amount of food [14, 54].

The importance of LOC eating as a key component of binge eating in adolescence was also reflected in our findings regarding correlates. Firstly, dose-response relationships were observed with the frequency of compensatory behaviors and risk of concurrent factors, with lowest rates or effect sizes observed for overeating, then for LOC eating and the highest for binge eating. Secondly, some preceding factors, such as emotional overeating and food enjoyment were most predictive for LOC eating compared to binge eating and overeating. Thirdly, emotional and behavioral problems, more unhealthy eating behaviors, body dissatisfaction and higher BMI, FMI and FFMI at 10 years were associated with LOC eating and binge eating, and less consistent associations were observed with overeating. These findings suggest that LOC eating is the most salient component of binge eating, as was previously also suggested by studies that compared overeating with binge eating [28, 29, 55].

Compensatory behaviors focusing on restrictive food intake, such as meal skipping, were more frequently observed in adolescents with subclinical binge eating symptoms compared to no symptoms. This finding is congruent with previous literature [56, 57], and expresses the difficulty of maintaining rigid food restriction that eventually seems to result in disinhibited eating. The observed association between restrained eating at 10 years and subsequent LOC eating and binge eating provides further evidence that this association already develops at an early age. Adolescents in our sample rarely endorsed severe compensatory behaviors that are indicative of BN or AN (e.g. purging and using weight loss medication), perhaps reflecting underreporting due to social desirability but likely also due to their young age. However, these extreme behaviors may increase throughout adolescence [58].

Almost no differences in demographic and parental characteristics according to subclinical binge eating symptoms were observed in this study. The higher prevalence of binge eating in girls in our study was in line with some previous literature [10, 21], but not all [1]. Ethnic and socioeconomic differences have been reported previously [10, 21], although some studies suggest that there are no consistent [30], or only rather small differences [31]. These latter studies are in line with our results, in which we observed that binge eating was slightly more prevalent among youth in both high and low household incomes. Further research, ideally in diverse samples with a large number of participants from all demographic levels, is needed to clarify the relationship between sociodemographic characteristics and binge eating symptoms.

Our results showed that subclinical binge eating symptoms are linked to a range of concurrent factors, including various eating behaviors such as emotional and restrained eating, body dissatisfaction, emotional and behavioral problems and higher BMI. These findings are in line with other studies that examined correlates of binge eating symptoms in adolescence, including eating behaviors, body dissatisfaction, overweight/obesity and psychiatric comorbidities [1, 13, 14, 21, 28, 59, 60]. The current study provided more insight, beyond these cross-sectional studies, into the temporal associations by showing that eating behaviors, body dissatisfaction, emotional and behavioral problems, and body composition were determinants of later binge eating symptoms.

Indeed, findings showed that preceding food approaching eating behaviors in childhood may be important precursors of subclinical binge eating symptoms in adolescence. This suggests that children who show high levels on a behavioral range scale of eating behaviors may be at greater risk of developing more severe symptomatology and even eating pathology later in life. Earlier studies also show that children with increasing levels of hedonic eating behaviors are at an increased risk of developing related eating pathology [4, 24]. Therefore, close monitoring of disordered eating behaviors in childhood may offer a window of opportunity for initiating early interventions if required.

We observed that the desire to be thinner at 10 years was associated with LOC eating and binge eating at 14 years, but not overeating. The absence of any sex differences in our study partially contradicts previous studies in which body dissatisfaction in early adolescence was associated with binge eating in late adolescence and (early) adulthood in females, but not males [29, 61]. These differences might be explained by the age differences between these studies and the current study but this requires further investigation.

While emotional and behavioral problems were cross-sectionally associated with binge eating symptoms in early adolescence, results were less conclusive for a temporal relationship. Here, adolescents with self-reported emotional and behavioral problems at 10 years were at higher risk for LOC eating and binge eating, but not overeating. However, when mothers reported on emotional and behavioral problems of their children, only associations between emotional problems and LOC eating and of behavioral problems with overeating were found. Prospective studies in adolescents indeed suggest that negative affect, anxiety, stress and attentional impulsivity may predict binge eating symptoms [19, 29, 62, 63]. Moreover, the direction of effects between binge eating symptoms and depression may differ by type of symptom: a bi-directional association of depressive symptoms with overeating and binge eating was observed in previous research over a one-year period, while a unidirectional association was observed from depressive symptoms towards LOC eating [64]. Our findings add to this that self-reported emotional problems, including depressive symptoms, in childhood may heighten the risk of LOC eating and binge eating, but not overeating.

This study showed that adolescents with a higher weight status (BMI, FMI and FFMI) in childhood were at heightened risk for LOC eating and binge eating. Although it is well-known that binge eating and obesity are linked [13], this study provides more insight in the potential directionality by showing that a higher BMI may be a precursor for later binge eating. Previous studies showed that a high BMI in childhood was associated with higher levels of food approach eating behaviors and disordered eating behaviors at later ages, suggesting that excess weight might upregulate appetite or increase reward sensitivity [65–67]. Furthermore, genetic vulnerability for anthropometric traits might also play a role, as polygenic risk scores for body composition (both fat mass and fat free mass) were positively associated with binge eating [68]. This suggests that BMI might be an important predictor of disordered eating behaviors and pathology, adding further evidence to the significance of maintaining a healthy weight and establishing healthy lifestyle habits throughout childhood and adolescence.

### Strengths and limitations

This study was able to differentiate between adolescents who experienced overeating only, LOC eating only and binge eating. This study was further strengthened by the large, prospective cohort of 14-year old adolescents which enabled us to explore the association between many potential determinants at an earlier time point and later subclinical binge eating symptoms.

The present study was limited by the assessment of BED symptoms, for which only two items were used. Therefore, we were not able to examine the prevalence and correlates for BED diagnosis and symptom severity. Furthermore, only adolescents self-reported on their binge eating symptoms while the use of multiple informants or a clinical interview could be preferred [26]. For instance, female adolescents were more likely to self-report binge eating symptoms than boys, while parental reports on adolescent binge eating symptoms, showed an almost equal distribution of binge eating among boys and girls [69]. Limitations were also present regarding the assessment of demographic and parental characteristics: household income and maternal education were assessed when the participants were six years old, which might have changed over time. Furthermore, comparison of our study sample with the initial Generation R sample (Additional file 1: Table S1) suggests a selection towards a slightly advantaged, more healthy sample. As such, the level of depressive symptoms among parents was rather low, which might be the reason why we did not observe any differences with subclinical binge eating in the offspring. Moreover, lifetime history of maternal eating disorders did not include BED, because at the time of assessment this was not an independent diagnosis in the DSM IV. Therefore, these results should be interpret with caution.

## Conclusion

Subclinical binge eating symptoms were common in 14-year old adolescents, of which LOC eating was most prevalent. Considering the high prevalence of LOC eating, and the overlapping preceding and concurrent factors of LOC eating and binge eating compared to overeating, LOC eating seems the most important component of binge eating. Food approaching eating behaviors, body dissatisfaction, more emotional and behavioral problems, and higher body composition were potential determinants of binge eating symptoms. Thus, prevention strategies could focus on LOC eating and its risk factors to interrupt the development of binge eating.

## Supplementary Information

**Additional file 1.** Supplement Subclinical binge eating symptoms.

## Data Availability

Data described in the manuscript, code book, and analytic code can be made available upon request to datamanagementgenr@erasmusmc.nl and will be discussed in the Generation R Study Management Team.

## Abbreviations

AN: Anorexia nervosa
BED: Binge eating disorder
BMI: Body Mass Index
BN: Bulimia nervosa
BSI: Brief symptom inventory
CBCL: Child behavior checklist
CI: Confidence interval
DAWBA: Development and Wellbeing Assessment
DEBQ: Dutch Eating Behavior Questionnaire
FMI: Fat Mass Index
FFMI: Fat Free Mass Index
LOC eating: Loss of control eating
OR: Odds ratio
SD: Standard deviation
TFEQ: Three Factor Eating Questionnaire
YSR: Youth self report
