# Supplementary material for: Subclinical binge eating symptoms in early adolescence and its preceding and concurrent factors: a population-based study
Source: J Eat Disord. 2022 Nov 23;10:180. doi: 10.1186/s40337-022-00688-6 (PMC9685858; doi:10.1186/s40337-022-00688-6)
Supplement: Supplementary file 1 — Additional file 1. Supplement Subclinical binge eating symptoms. [file 40337_2022_688_MOESM1_ESM.docx]

| **Supplementary Table 1. Demographic characteristics of the study sample, participants who were lost to follow up and the total Generation R sample** | | | | | | |
| --- | --- | --- | --- | --- | --- | --- |
|  | **Study sample**  **(n=3595)** | | **Sample excluded due to missing data on subclinical binge eating symptoms^a^**  **(n=3247)** | | **Total Generation R sample (live births)**  **(n= 9749)** | |
| **Demographic and child characteristics** | **n** | **Mean/median/**  **% (95% CI)** | **n** | **Mean/median/% (95% CI)** | **n** | **Mean/median/% (95% CI)** |
| Age at outcome assessment (years), | 3595 | 13.88 (13.87, 13.90) | 224 | 14.19 (14.09, 14.29) |  |  |
| Sex | 3595 |  | 3247 |  | 9746 |  |
| Girls | 1896 | 52.7 (51.1,54.6) | 1506 | 46.4 (44.8, 48.0) | 4808 | 50.7 (49.8, 51.6) |
| Boys | 1699 | 47.3 (45.4, 48.9) | 1741 | 53.6 (52.0, 55.2) | 4938 | 49.3 (48.4, 50.3) |
| Ethnicity | 3566 |  | 3072 |  | 9092 |  |
| Dutch | 2444 | 68.5% (67.0, 70.1) | 1542 | 50.2 (48.4, 54.9) | 3392 | 53.8 (52.9, 54.8) |
| Other western | 313 | 8.8 (7.8, 9.6) | 229 | 7.5 (6.5, 8.4) | 805 | 8.9 (8.3, 9.4) |
| Non-western | 809 | 22.7 (21.1, 24.0) | 1301 | 42.4 (40.7, 44.1) | 4895 | 37.3 (36.3, 38.3) |
| Household income | 3191 |  | 2015 |  |  |  |
| High (>4000 euros per month) | 1292 | 40.5 (38.7, 42.2) | 582 | 28.9 (27.0, 30.9) |  |  |
| Medium (1600-4000 euros per month) | 1587 | 49.7 (47.9, 51.6) | 971 | 48.2 (46.0, 50.2) |  |  |
| Low (< 1600 euros per month) | 312 | 9.8 (8.8, 10.9) | 462 | 22.9 (21.0, 24.7) |  |  |
| BMI SD score at 14 years | 3286 | 0.15 (0.11, 0.19) | 1633 | 0.46 (0.40, 0.52) |  |  |
| Mother-reported emotional problems | 3458 | 4.00 (4.00, 4.00) | 1264 | 4.00 (4.00, 5.00) |  |  |
| Mother-reported behavioral problems | 3452 | 2.00 (2.00, 2.00) | 1258 | 3.00 (3.00, 4.00) |  |  |
| **Parental characteristics** |  |  |  |  |  |  |
| Maternal educational level | 3329 |  | 2164 |  |  |  |
| High (higher vocational education to university) | 2195 | 65.9 (64.2, 67.5) | 1061 | 49.0 (46.9, 51.2) |  |  |
| Medium (lower vocational education) | 880 | 26.4 (25.1, 28.1) | 753 | 34.8 (32.8, 37.0) |  |  |
| Low (no education to high school) | 254 | 7.6 (6.7, 8.6) | 350 | 16.2 (14.6, 17.6) |  |  |
| Maternal history of an eating disorder | 3417 |  | 2575 |  | 7654 |  |
| Yes | 319 | 9.3 (8.3, 10.3) | 251 | 9.7 (8.5, 10.9) | 727 | 9.5 (8.8, 10.2) |
| No | 3098 | 90.7 (89.7, 91.7) | 2324 | 90.3 (98.1, 91.5) | 6927 | 90.5 (89.8, 91.2) |
| Maternal pre-pregnancy BMI | 3192 | 24.31 (24.17, 24.44) | 2949 | 25.17 (25.00, 25.34) | 8753 | 24.89 (24.79, 24.98) |
| Paternal BMI during pregnancy | 2732 | 25.21 (25.09, 25.34) | 2029 | 25.31 (25.17, 25,47) | 6320 | 25.30 (25.22, 25.39) |
| Maternal depressive symptoms | 3138 | 0.00 (0.00, 0.00) | 1517 | 0.00 (0.00, 0.00) | 4864 | 0.00 (0.00, 0.00) |
| Paternal depressive symptoms | 2600 | 0.00 (0.00, 0.00) | 877 | 0.00 (0.00, 0.00) | 3558 | 0.00 (0.00, 0.00) |
| 95% confidence intervals were obtained by using bootstrapping (results of 1000 samples). ^a^ This group consists of adolescents who provided consent for the 14-years wave, but did not report on subclinical binge eating symptoms. | | | | | | |

| **Supplementary Table 2. Frequency of binge eating symptoms in 14 year old adolescents.^1^** | | |
| --- | --- | --- |
|  | **Binge Eating Symptoms** | |
|  | **Overeating** | **LOC eating** |
| **Frequency of symptoms in the past 3 months** | **n (%)** | **n (%)** |
| **Total** |  |  |
| Did not happen | 3395 (94.4) | 3264 (90.8) |
| Less than once per month | 89 (2.5) | 198 (5.5) |
| 1 or more times per month | 84 (2.3) | 106 (2.9) |
| 1 or more times per week | 27 (0.8) | 27 (0.8) |
| **Males** |  |  |
| Did not happen | 1629 (95.9) | 1572 (92.5) |
| Less than once per month | 30 (1.8) | 76 (4.5) |
| 1 or more times per month | 31 (1.8) | 38 (2.2) |
| 1 or more times per week | 9 (0.5) | 13 (0.8) |
| **Females** |  |  |
| Did not happen | 1766 (93.1) | 1692 (89.2) |
| Less than once per month | 59 (3.1) | 122 (6.4) |
| 1 or more times per month | 53 (2.8) | 68 (3.6) |
| 1 or more times per week | 18 (0.9) | 14 (0.7) |
| ^1^ The frequency of each binge eating symptom separately is presented without combining the symptoms. | | |

| **Supplementary Table 3. Univariate associations between concurrent or preceding factors and binge eating symptoms in the past three months in early adolescence at 14 years, stratified by child sex.** | | | | | | |
| --- | --- | --- | --- | --- | --- | --- |
|  |  |  | **Binge Eating Symptoms** | | | |
|  |  |  | **No symptoms** | **Overeating only** | **LOC eating only** | **Binge eating (overeating with LOC eating)** |
| **Co-occurring factors** |  | **Total n** |  | **OR (95% CI)** | **OR (95% CI)** | **OR (95% CI)** |
| Restrained eating | Girls | 1888 | Ref. | 1.31 (1.07, 1.61) | 1.58 (1.37, 1.82) | 2.23 (1.81, 2.74) |
|  | Boys | 1686 | Ref. | 1.24 (0.94, 1.63) | 1.37 (1.15, 1.64) | 1.12 (0.73, 1.71) |
| Self-reported behavioral problems | Girls | 1831 | Ref. | 2.20 (1.78, 2.71) | 1.78 (1.51, 2.10) | 2.56 (2.03, 3.23) |
|  | Boys | 1636 | Ref. | 1.25 (0.96, 1.63) | 1.32 (1.11, 1.57) | 1.62 (1.23, 2.14) |
| **Preceding factors** |  |  |  |  |  |  |
| BMI SD score | Girls | 1761 | Ref. | 1.35 (1.06, 1.72) | 1.42 (1.18, 1.70) | 2.09 (1.57, 2.77) |
|  | Boys | 1568 | Ref. | 1.00 (0.74, 1.34) | 1.25 (1.01, 1.55) | 1.06 (0.67, 1.68), |
| Fat Mass Index SD score | Girls | 1747 | Ref. | 1.17 (0.89, 1.53) | 1.37 (1.14, 1.64) | 1.88 (1.48, 2.40) |
|  | Boys | 1547 | Ref. | 1.11 (0.81, 1.52) | 1.32 (1.08, 1.62) | 0.87 (0.48, 1.55) |
| Fat Free Mass Index SD score | Girls | 1747 | Ref. | 1.39 (1.09, 1.78) | 1.31 (1.09, 1.57) | 1.67 (1.29, 2.16) |
|  | Boys | 1547 | Ref. | 0.88 (0.64, 1.21) | 1.16 (0.93, 1.44) | 1.18 (0.72, 1.92) |
| *All factors are standardized. Results were obtained with multinomial logistic regression analysis and all associations were adjusted for exact age at outcome assessment. LOC: loss of control, OR: Odds Ratio, CI: Confidence Interval, SD: Standard Deviation | | | | | | |
